# PAC1- and VPAC2 receptors in light regulated behavior and physiology: Studies in single and double mutant mice

**DOI:** 10.1371/journal.pone.0188166

**Published:** 2017-11-20

**Authors:** Jens Hannibal, Birgitte Georg, Jan Fahrenkrug

**Affiliations:** Department of Clinical Biochemistry, Faculty of Health Sciences, Bispebjerg Hospital, University of Copenhagen, Copenhagen, Denmark; Kent State University, UNITED STATES

## Abstract

The two sister peptides, pituitary adenylate cyclase activating polypeptide (PACAP) and vasoactive intestinal polypeptide (VIP) and their receptors, the PAC1 –and the VPAC2 receptors, are involved in regulation of the circadian timing system. PACAP as a neurotransmitter in the retinohypothalamic tract (RHT) and VIP as a neurotransmitter, involved in synchronization of SCN neurons. Behavior and physiology in VPAC2 deficient mice are strongly regulated by light most likely as a result of masking. Consequently, we used VPAC2 and PAC1/VPAC2 double mutant mice in comparison with PAC1 receptor deficient mice to further elucidate the role of PACAP in the light mediated regulation of behavior and physiology of the circadian system. We compared circadian rhythms in mice equipped with running wheels or implanted radio-transmitter measuring core body temperature kept in a full photoperiod ((FPP)(12:12 h light dark-cycles (LD)) and skeleton photo periods (SPP) at high and low light intensity. Furthermore, we examined the expression of PAC1- and VPAC2 receptors in the SCN of the different genotypes in combination with visualization of PACAP and VIP and determined whether compensatory changes in peptide and/or receptor expression in the reciprocal knockouts (KO) (PAC1 and VPAC2) had occurred. Our data demonstrate that in although being closely related at both ligand and receptor structure/sequence, PACAP/PAC1 receptor signaling are independent of VIP/VPAC2 receptor signaling and vice versa. Furthermore, lack of either of the receptors does not result in compensatory changes at neither the physiological or anatomical level. PACAP/PAC1 signaling is important for light regulated behavior, VIP/VPAC2signaling for stable clock function and both signaling pathways may play a role in shaping diurnality versus nocturnality.

## Introduction

In mammals, the brain’s biological clock located in the suprachiasmatic nucleus (SCN) [1] generates circadian rhythms in physiology and behavior of approximately 24 h. 10–20.000 neurons constitute the neuronal network of the SCN, and the individual neurons express a molecular clockwork based on positive–and negative feedback loops of so-called clock proteins and their respective genes [2]. To maintain a rhythmic clock function, the individual neurons in the SCN need to be synchronized. Important signals are mediated by the neuropeptide VIP and its receptor, the VPAC2 receptor [3–5]. VIP is expressed in neurons of the ventral SCN while the VPAC2 receptors occur throughout the SCN although with higher expression corresponding to the dorsomedial shell region of the SCN [6, 7]. Mice lacking either VIP or VPAC2 receptors have similar phenotype. Both strains of mice become arrhythmic in constant darkness but show stable nocturnal activity during normal light-dark photoperiods. The nocturnal behavior is strongly dependent on the light conditions [4, 5, 8] as are other rhythmic behaviors and physiology such as temperature, metabolism, food intake, heart rate and hormone secretion. However, in VIP/VPAC2 receptor deficient mice these rhythms are significantly advanced by 5–6 h compared to activity onset [5, 9–11]. The ventral SCN receives retinal input via the retinohypothalamic tract (RHT) which daily entrains the clock to light [12, 13], and VIPergic neurons located in this part of the SCN project both within and outside the nucleus [6]. The VIPergic neurons are hypothesized to be important for relaying photic information in light regulated behavior [14].

The neuropeptide PACAP is structurally related to VIP. Three seven transmembrane G-protein coupled receptors (GPCR) for PACAP have been identified–PAC1, VPAC1 and VPAC2; however, only the PAC1 receptor is selective for PACAP, while VPAC1 and VPAC2 receptors show equal affinities for PACAP, and VIP [15]. PACAP has been attributed a role as neurotransmitter in the RHT involved in masking control [16–18]. PACAP signaling is mediated by PAC1 receptors located in SCN neurons [17] and mice lacking either PACAP or PAC1 receptors display impaired photoentrainment and masking at low light intensities [16–18].

Behavior and physiology in VPAC2 deficient mice are strongly regulated by light most likely as a result of masking [3]. Consequently, we decided to use VPAC2 and PAC1/VPAC2 double mutant mice in comparison with PAC1 receptor deficient mice to further elucidate the role of PACAP in the light mediated regulation of behavior and physiology of the circadian system. We compared circadian rhythms in mice equipped with running wheels or implanted radiotransmitter recording core body temperature, which were kept in a FPP and SPP at high and low light intensity. Furthermore, we examined the expression of PAC1- and VPAC2 receptors in the SCN of the different genotypes in combination with visualization of PACAP and VIP and determined whether compensatory changes in peptide and/or receptor expression in the reciprocal knockouts (KO) (PAC1 and VPAC2) had occurred.

## Material and methods

### Animals

From our colony of PAC1 receptor deficient [16] and VPAC2 deficient mice [5] we generated a strain of PAC1/VPAC2 deficient double mutant mice. Male and female mice of each genotype were included in the study. Mice were 10–12 weeks old when included in the experiments and were maintained in 12:12 h LD cycles in individual cages with water and food *ad libitum* (Altromin 1324; Altromin Spezialfutter, Germany) unless otherwise stated. Animals were treated according to the principles of Laboratory Animal Care (Law on Animal Experiments in Denmark, publication 1306, November 23, 2007) and Dyreforsoegstilsynet, Ministry of Justice, Denmark, who issued the license number 2008/561-1445 to Jan Fahrenkrug and thereby approving the study.

### Circadian behavior

#### Measurements of running wheel activity rhythms

Wild-type and KO mice (eight animals of each genotype, four males and four females) were transferred to individual cages equipped with a running wheel in ventilated, light-tight chambers with controlled white lighting (5–300 lux). Wheel running activity was monitored by an on-line PC connected via a magnetic switch to the MiniMitter Running Wheel activity system (consisting of QA-4 activity input modules, DP-24 dataports and Vital View data acquisition system, MiniMitter Company, Inc. Sunriver, OR, USA vers. 4.1) [19]. Wheel revolutions were collected continuously in 6 min bins. Animals were entrained to a 12:12 light/dark (LD) cycles (lights on at 7:00 a.m. designated Zeitgeber time (ZT) = 0, off at 7.00 p.m. = ZT12) for at least 14 days before start of experiments.

#### Measurements of core body temperature

Core body temperature was monitored by a radiotransmitter device (G2 E-mitter; MiniMitter; Sunriver, OR, USA) implanted in the abdominal cavity of each animal (six animals of each genotype, three males and three females) by sterile techniques (see E-Mitter Implantation Procedure, part number 910-0014-05 Rec C, Respironics, MINI MITER) under general anaesthesia (a subcutaneous injection of a mixture of fetanyl (0.20 mg/kg body weight (BW), fluanisone (6.25 mg/kg BW), and midazolam (3.13 mg/kg BW)). After operation, the mice received antibiotic treatment by a single dose of ampicillin. Postoperative pain was reduced by a single subcutaneous injection of 5 mg/kg BW of carprofen. Radiosignals were recorded by a receiver board (ER-4000 energizer receiver) below each cage housing an animal and stored via Vital View data acquisition system (MiniMitter Company, Inc. Sunriver, OR, USA vers. 4.1) on a PC in 6 min bins. The mice were allowed to recover from surgery for at least two weeks before attending the experiments. The ambient temperature within the cabin which stored 12 cages was 21–22°C.

#### Light source and intensity measurements

White lightning was delivered from fluorescent tubes placed above each cage. The light intensity was adjusted by a resistance. Light intensity was measured using an Advantest Optical Power meter TQ8210 (MetricTest, Hayward, CA), and measurements were determined at settings of 514 nm; 300 lux (115.0 μW/cm^2^) and 10 lux (4.3 μW/cm^2^), respectively.

#### Running wheel behavior FPP and SPP

PAC1KO, VPAC2KO and double mutant and their corresponding wild type mice were examined during a FPP of 12:12 h LD cycles and a SPP regime of 1:11LD, 1:11LD using light intensities of 300 and 10 lux. All animals were kept in LD for at least 10 days followed by a SPP in which light was turned on at ZT0 and at ZT11 and turned off at ZT1 and ZT12.

#### Core body temperature during FPP and SPP

Core body temperature (TP) was measured in PAC1KO, VPAC2KO, double mutant and wild type mice at similar light conditions as mentioned above.

### Data analysis

Data obtained from the Minimitter Running Wheel activity system and the ER-4000 energizer receiver system were analysed in ClockLab (ActiMetric Software, Coulbourn Instruments, Wilmette, IL, USA) running under Matlab (v. R2012a, 64-bit for Windows7, MathWorks, Natick, MA, USA) environment. Data in the diagrams are the average of five days activity from each animal (n = 6–8) obtained after the animal had adapted to the light conditions and presented as mean ± SEM. Data from 5 days from each animal were averaged and plotted as activity/temperature cycle using GraphPad Prism 5.0.

### Statistics

Statistics were performed using GraphPad Prism 5.0. For comparison of two independent groups, Mann Whitney U test was used. For the comparison of light responsiveness between two genotypes during 24 cycles of either LD or SPP two-way ANOVA followed by the Bonferroni posttest were performed. *P*< 0.05 was considered statistically significant.

### Generation of PAC1 and VPAC2 antibodies

The antibodies against the PAC1 and VPAC2 receptors were raised in rabbits immunized with recombinant fusion proteins expressed in E. coli BL21(DE3)pLysS and purified as described previously [20].

For production of PAC1 antibodies the following E.coli produced amino acid sequence (PRO6095) was used for immunization:

*MRGSHHHHHHGMASMTGGQQMGRDLYDDDDKDHPFT*MARVLQLSLTALLLPVAIAMHSDCIFKKEQAMCLERIQRANDLMGLNESSPGCPGMWDNITCWKPAQVGEMVLVSCPEVFRIFNPDQVWMTETIGDSGFADSNSLEITDMGVVGRNCTEDGWSEPFPHYFDACGFDDYEPESGDQDYY**FNNFTVSFWLRVPKVSASHLE**KRKWRSWKVNRYFTMDFKHRHPSLASSGVNGGTQLSILSKSSSQLRMSSLPADNLAT. A His-tag used for purification is shown in italics. The underlined sequences are the N-terminal and C-terminal parts of the rat PAC1 receptor, respectively. The sequence in bold in between the two is the tetanus toxin p30 epitope.

For VPAC2 immunization the sequence (PRO6083) was used: *MRGSHHHHHHGMASMTGGQQMGRDLYDDDDKDHPFT*MRASSVVLTCYCWLLVRVSSIHPECRFHLEQEEETKCAELLSSQMENHRACSGVWDNITCWRPADIGETVTVPCPKVFSNFYSRPGNISKNCTSDWSETFDFIDACGYNDPEDGKIT**FFNNFTVSFWLR**RAQGERQPPGA. As for PAC1, the His-Tag is shown in italics. The underlined sequences are the N-terminal parts of the rat VPAC2 receptor. The sequence in bold is part of the tetanus toxin p30 epitope, the rest of the sequence shown is nonsense due to a frame shift error. Recombinant expression, purification and immunizations of rabbits were performed as described previously [20].

Antisera from the rabbits with the code numbers 35J8 (PAC1) and 623S (VPAC2) were selected for use after characterization (see below). Chinese hamster ovary (CHO) cells were stably transfected with expression plasmids harboring the cDNA of either rat PAC1 (BG30-1), rat VPAC2 (BG11-1), or the empty vector (pcDNA3). The latter served as negative control. CHO-PAC1 (81–13) and CHO-VPAC2 (80–5) stably expressing PAC1 and VPAC2 receptor, respectively, or pcDNA3 (CHO-pcDNA3) were obtained by G418 selection and serial dilution. Identification of high expressing cell lines was done by Northern blotting. The CHO cells were used as part of the antibody characterization.

### Immunohisto/cytochemistry and antibody characterization

The PAC1 and the VPAC2 receptor antibodies were characterized by immunocytochemical staining of CHO cells expressing the respective receptor, and by staining mouse brain sections from wild type and PAC1—and VPAC2 deficient mice. Furthermore, immunoblotting was performed on extracts of the above mentioned cell lines. For immunohistochemistry mice were perfused with Stefanini fixative as described previously [19]. For immunocytochemistry, cells were fixed in Stefanini fixative for 30 min. Brain sections and cells were treated by antigen retrieval solution for 16 h at 40°C or 80°C for 1.5 h (DAKO ChemMate, Glostrup, Denmark, code No. S 203120 in distilled water, pH 6) before processing for immunohistochemistry. Sections and cells were subsequently incubated (the primary antibodies (PAC1 code no. 35J8 diluted 1:10,000, VPAC2 code 623S, diluted 1:20,000) 2–4 d at 4°C and visualized using the Envision (code K4002, dilutes 1:2, DAKO) and tyramide conjugated Alexa488 (Molecular Probes). PACAP immunoreactivity was visualized using a rabbit anti-PACAP antibody (code 523C, diluted 1:160,000; RRID: AB_2650426) characterized in details previously [21]. This antibody was raised against PACAP38 and recognizes an epitope between amino acid 16–38 of PACAP38 and shows no cross reactivity with VIP or other related peptides [21]. No staining was observed in brain sections from PACAP KO mice (data not shown). The rabbit anti-VIP antibody (code no. 291E, diluted 1:1,000, RRID: AB_2313759) has been characterized in details previously [22]. For visualization of either PACAP and PAC1 receptors or VIP and VPAC2 receptors, we used the method previously described on two primary antibodies raised in the same species [23] using biotinylated tyramide (tyramide system amplification; DuPont NEN, Boston, MA), and streptavidin-conjugated AlexaFluor dyes and/or Envision (Dako, ChemMate, Glostrup, Denmark) and tyramide-conjugated Alexa dyes. In double staining of VPAC2/VIP, VPAC2 was visualized by Envision and VIP by a secondary anti-rabbit antibody conjugated to Alexa594 (A21207, Thermo-Fisher Scientific, Rockford, USA.

Photomicroscopy was performed using an iMIC confocal microscope equipped with the following objectives: X10, numerical aperture (NA) = 0.35; X20, NA = 0.75; X40, NA = 1.3 and X60, NA = 1.46. Using the X60, the highest resolution ((r = λ/NA), where λ is the imaging wavelength) was for X60 = 174 nm. Resolution in the z-axis was at X60 0.2 μm. All images in Z-stacks photographed using the X40 or X60 objective were deconvoluted in AutoQuantX, version 3.04 (Media Cybernetics, Inc. Rockville, USA) before analyzed in IMARIS vers. 8.4.1 (RRID:SCR_007370) from Bitplane, Switzerland (http://www.bitplane.com). For 3D analysis at high magnification (X60), images obtained by confocal microscopy, typically 60–80 images separated in the Z-level by 0.2 μm, were deconvolved in AutoQuant X (Version 3.02, Media Cybernetics, Inc, Rockville, USA). Co-localization and analysis of close apposition (synaptic appositions), Z-stacks in 3D reconstructions obtained by X60 objective were analysed in the co-localization module of IMARIS. Co-localization was established if two colours representing the different antigens were found in the same pixel. In X60, one pixel had a dimension of 100 x 100 nm. In cases where PACAP or VIP were co-localized with PAC1- or, VPAC2-immunoreactivity in anatomical structures compatible with the localization of synapses, the co-localization most likely represents genuine synaptic appositions.

All images were adjusted for brightness and contrast either in Fiji or in Photoshop CS5 (Adobe, San Jose, CA, RRID:SCR_014199) and mounted into plates in Adobe Illustrator CS5 (Adobe).

#### Immunoblotting

The CHO-PAC1 (81–13), CHO-VPAC2 (80–5) and pcDNA3 (CHO-pcDNA3) cells were mechanically disrupted in: 25 mM HEPES (N-[2-hydroethyl]piprazine-N’[2-ethanesulfonic acid]) pH: 7.4 containing 50 mg/L bacitracin using a polytron. Homogenate were spun for 20 min at 27,000 x g, 4°C and the pellets were suspended, homogenized and spun again whereafter the new pellets were suspended in SDS-sample buffer containing 8% SDS (sodium dodecyl sulfate), 24% glycerol, 100 mM Tris-Cl pH 6.8, 40 mM dithiothreitol, 5 mM EDTA (ethylenediaminetetraacetic acid disodium salt dehydrate) and 0.025% Coomasie Brilliant Blue. After denaturation and before application on gels, the samples were added iodoacetamide to a final concentration of 170 mM and incubated for 20 min at room temperature. The samples and SeeBlue Markers (ThermoFisher Scientific) were electrophoresed on NuPage 4–12% Bis-Tris gels (NP0323, ThermoFisher Scientific) and transferred to PVDF (polyvenylidendiflurid) membranes (ThermoFisher Scientific) by semidry blotting. The membranes were blocked with 3% dry non-fat milk in Tris buffer saline (20 mM Tris-Cl, 140 mM NaCl, pH 7.6). The primary antibodies directed against the PAC1 and VPAC2 receptors were diluted 1:1000. Swine anti-rabbit IgG Horseradish Peroxidase conjugated antibodies (P0399, Agilent) diluted 1:2500 were used for detection using enhanced chemiluminescence (ECL, Pierce) and exposed to Hyperfilm ECL (GE Healthcare Life Sciences).

## Results

### Animals

We found no signs of reduced fertility or gross anatomical abnormality in any of the mutant mice, in addition no sex differences were observed in any of the experiments.

### Locomotor behavior in PAC1-, VPAC2- and PAC1/VPAC2 deficient mice during FPP, constant darkness (DD) and SPP

Running wheel (RW) activity and TP during two different light regimes were compared between single mutant–and double mutant mice.

**Fig 1:**
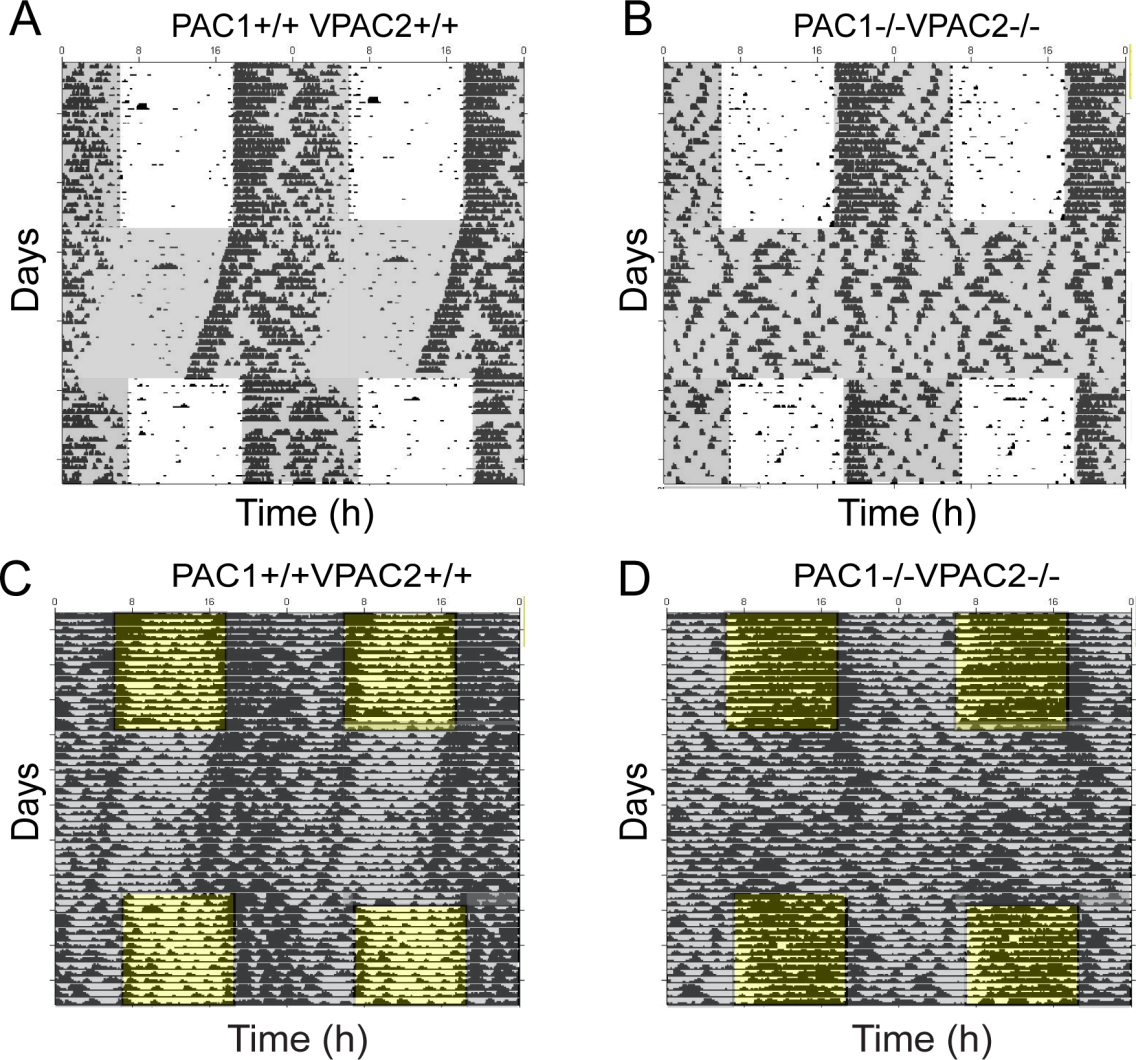
Locomotor activity and core body temperature (TP) in PAC1+/+VPAC2+/+ and PAC1-/-VPAC2-/- mice. (**A-B**) Representative double-plotted wheel-running actograms of PAC1+/+VPAC2+/+ and PAC1-/-VPAC2-/- males during a 24 h LD cycle in 300 lux followed by free-running in constant darkness (indicated by shading). (**C-D**) Representative double-plotted actograms of core body temperature recorded by telemetry in PAC1+/+VPAC2+/+ and PAC1-/-VPAC2-/- males during a 24 h LD cycle followed by free-running in constant darkness (indicated by shading). Note that PAC1-/-VPAC2-/- mice loose rhythmicity during constant darkness (**B** and **D**). Note that PAC1-/-VPAC2-/- mice are arrhythmic as determined by telemetry and that TP start raising in the morning almost 180 degrees out of phase with wild type animals during LD (**D**). The black/yellow bar in C and D represents the LD period.

**Fig 2:**
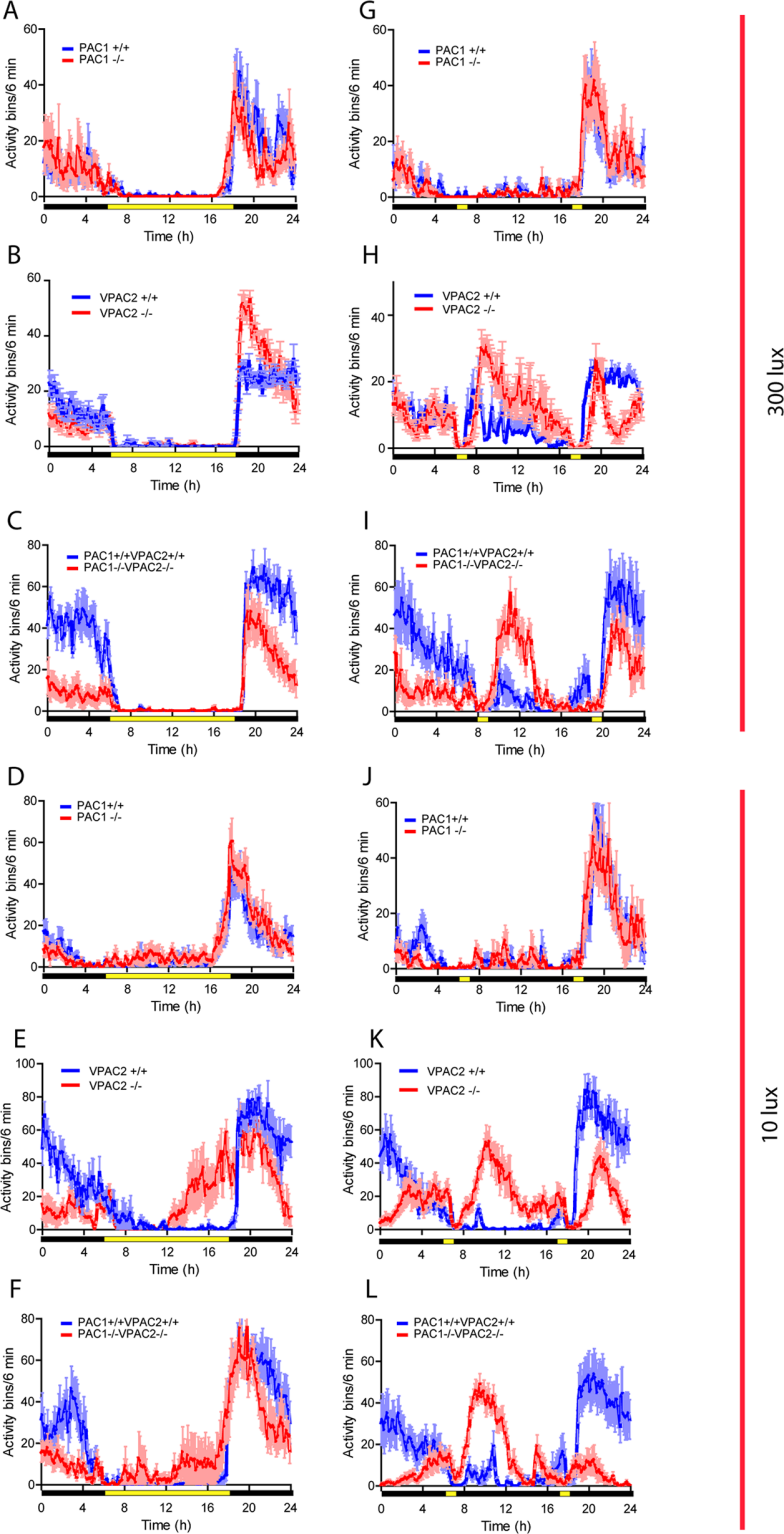
Running wheel activity in mice lacking the PAC1 (PAC1-/-), - the VPAC2 (VPAC2-/-) and both (PAC1-/-VPAC2-/-) receptors (red) and their respective wild type (blue) during parametric a (LD cycle) and non-parametric light conditions (SP) during light intensity of 300 and 10 lux. Each diagram is an average of five days activity from each animal (n = 8 in each group, four males and four females) obtained after the animal has adapted to the light conditions and are presented as mean ±SEM.

During a full photoperiod of 12:12 h LD at 300 lux followed by a period of constant darkness (DD) we confirmed that PAC1 deficient mice entrained to the LD cycle and showed stable nocturnal activity during DD (see also Fig 2 in [19]). VPAC2 deficient mice demonstrated stable nocturnal locomotor activity and became immediately arrhythmic after transfer to DD (see also Fig 1 in [5]). Double mutant mice displayed as the VPAC2 deficient mice stable nocturnal running wheel activity during the LD cycle with onset corresponding to lights off similar to wild type mice. They also became arrhythmic from the first cycle in DD (Fig 1). PAC1KO and wild type mice demonstrate equal distribution and intensity of nocturnal activity (Fig 2A), while VPAC2KO mice ran significantly more during the first part of the subjective night compared to wild type mice (Fig 2B). Double mutant mice, on the other hand, ran significantly less than wild type mice during the dark period (Fig 2C). At low light intensity PAC1KO and wild type mice entrained to the LD cycle with low daytime activity and onset at lights off as previously reported (Fig 2D). VPAC2KO mice showed an advanced activity onset by nearly 6 h and displayed significant less activity at late night (Fig 2E). Double mutant mice behavior was similar to VPAC2KO, although the activity before the dark onset was lower and more activity appeared in the subjective night period (Fig 2F).

During SPP at 300 lux, PAC1KO and wild type mice entrained to the subjective night phase (Fig 2G). VPAC2- and double mutant mice on the other hand, displayed similar changes in activity as described previously for both VPAC2 [5], and VIP- [24]- deficient mice with a biphasic activity pattern aligning the first activity period to the end of the morning light pulse at ZT0, and the second activity period starting at the end of the evening light pulse (Fig 2H and 2I). Both VPAC2KO mice and double mutant mice had the majority of their activity during the first 6 h after the first light pulse (at ZT1) (Fig 2H and 2I).

During low light intensity, PAC1KO and wild type mice entrained to the SPP cycle with activity initiated after the light pulse at ZT12 (Fig 2J). The biphasic pattern of activity found at 300 lux was also observed at low light intensity in the VPAC2 mutant mice (Fig 2K). In contrast, double mutant mice displayed a “diurnal pattern” of activity initiated at the end of the morning light pulse (Fig 2L).

### Core body temperature in PAC1-, VPAC2- and PAC1/VPAC2 deficient mice during FPP, DD and SPP

**Fig 3:**
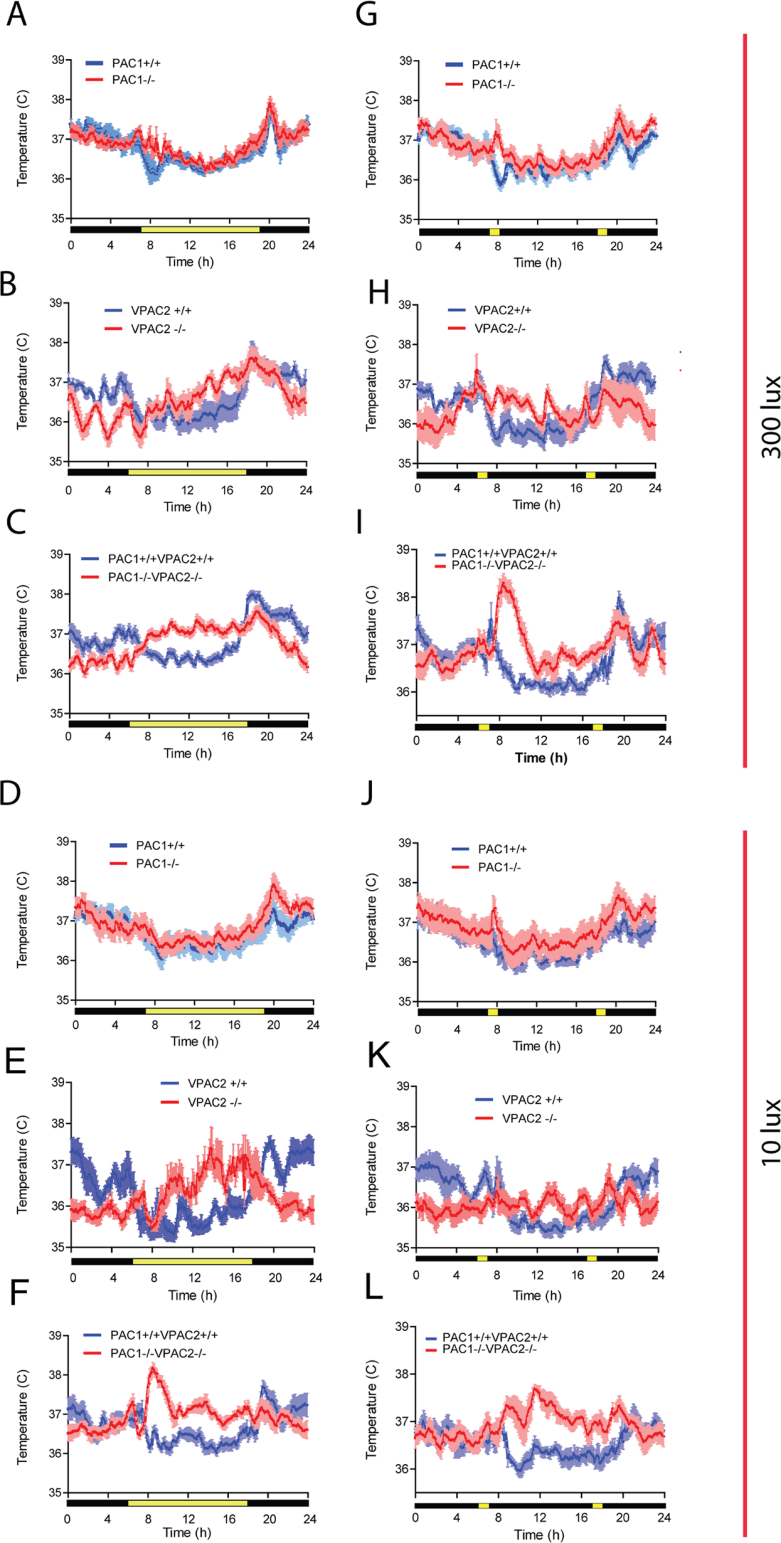
Core body temperature in mice lacking the PAC1 (PAC1-/-), - the VPAC2 (VPAC2-/-) and both (PAC1-/-VPAC2-/-) receptors (red) and their respective wild type (blue) during parametric a (LD cycle) and non-parametric light conditions (SP) during light intensity of 300 and 10 lux. Each diagram is an average of five days activity from each animal (n = 6 in each group, three males and three females) obtained after the animal has adapted to the light conditions and are presented as mean ±SEM.

To further characterize circadian physiology involving PACAP and VIP signaling, TP was recorded by intraperitoneal telemetry. During a full photoperiod of 12:12 h LD at 300 lux followed by a period of constant darkness (DD), we confirmed that PAC1 deficient mice entrained to the LD cycle (Fig 3A) and showed a stable rise in TP during DD (data not shown). VPAC2 deficient mice demonstrated a stable rise in TP which was advanced 6–8 h compared to wild type animals (Fig 3B) and TP rhythm was abolished in DD (see also Fig 5 in [5]). Double mutant mice displayed as the VPAC2 deficient mice a stable rise in TP during the FPP, which was advanced as observed in VPAC2 single mutant mice (Fig 3C) and the TP rhythm disappeared from the first cycle in DD (Fig 1D). During 10 lux at FPP PAC1KO and wild type mice displayed identical rise in TP as observed at 300 lux starting at the end of the subjective day peaking at early subjective night and declined to be lowest at subjective day (Fig 3A and 3D). The daily rhythm of TP was significantly changed in VPAC2- and in double mutant mice. Both VPAC2- and double mutant mice demonstrated "diurnal like" TP curves compared to wild type similar to that observed at the higher light intensity. In both genotypes, TP started to rise from dawn peaking during the subjective day whereafter TP gradually declined during the subjective night (Fig 3B, 3C, 3E and 3F). Double mutant mice deviated slightly compared to VPAC2 single mutant mice by having a marked increase in TP after morning lights off followed by a gradual decrease during the remaining day and night (Fig 3F).

During SPP at 300 and 10 lux, TP rhythms displayed similar profile as observed in FPP and no difference was found between PAC1KO—and wild type mice (Fig 3G and 3J). In VPAC2KO mice, TP peaked corresponding to the morning light pulse at 300 lux with a gradual decline during the subjective day but significantly higher compared to wild type animals (Fig 3H). Double mutant mice displayed a marked increase in TP immediately after the morning light pulse followed by a fall in TP over the following 6 h and a new but smaller rise in TP after the evening light pulse (Fig 3I). Although slightly more variable, the profiles from both VPAC2 –and double mutant mice at 10 lux were comparable to the TP during the full photoperiod at the lower light intensity and with that of SPP at 300 lux (Fig 3K and 3L).

### Characterization of PAC1 and VPAC2 antisera

See supporting information S1 File and S1 Fig.

### Localization of PACAP and PAC1 receptor in the mouse SCN

**Fig 4:**
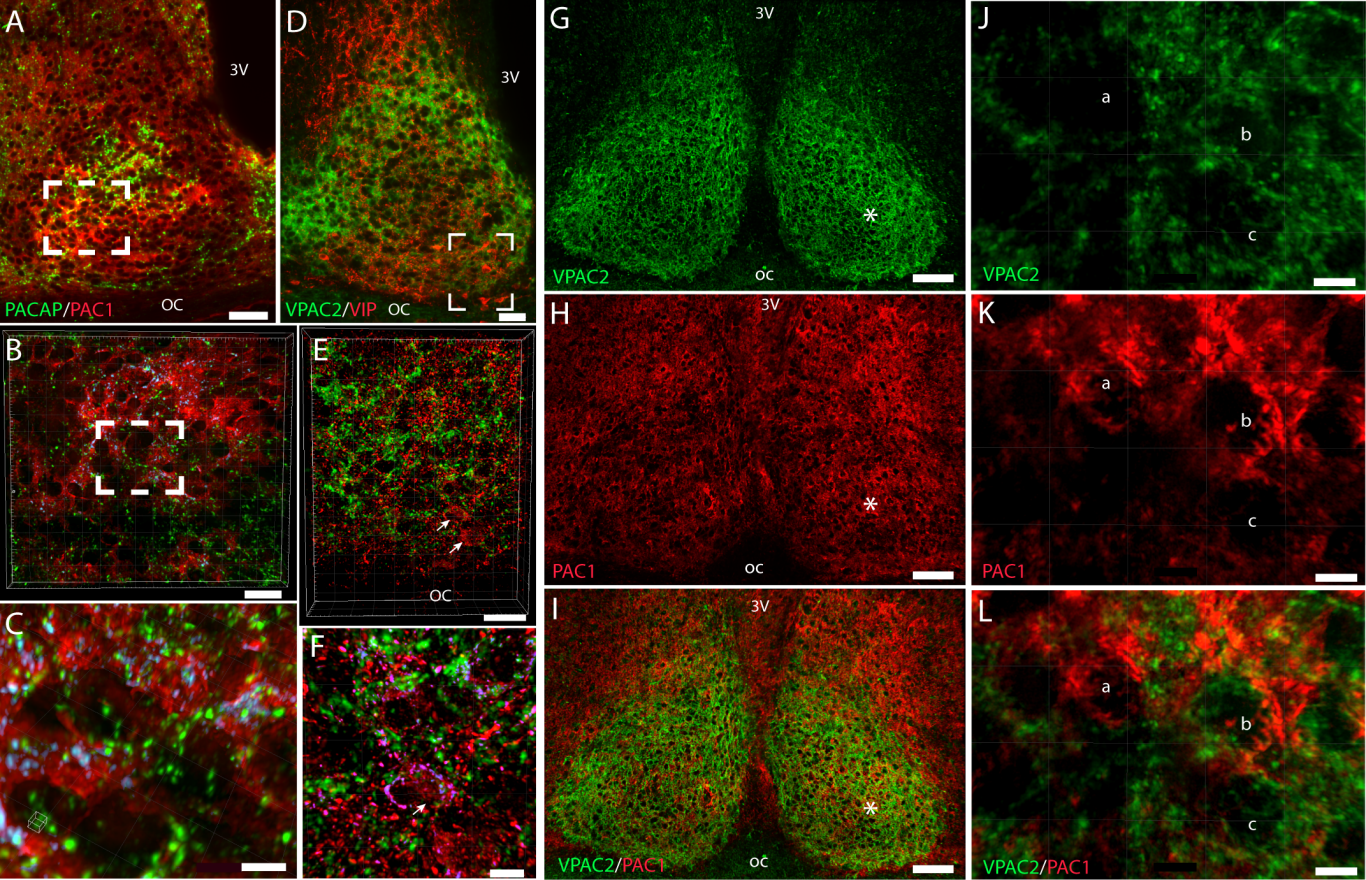
Distribution of PACAP (green) and PAC1 receptor (red) immunoreactivity (A-C), and VIP (red) and VPAC2 (green) immunoreactivity in the mouse wild type SCN (D-F). PACAP immunoreactive fibers from the eye are found in the retinorecipient part of the SCN in close apposition to the PAC1 receptor immunoreactivity (A). 47 digital sections (Z- stack) were used to make the 3D reconstruction seen in B-C. Imaris co-localization module (see material and methods) was used to determine close appositions (indicated blue in B-C) most likely representing synaptic appositions. C represents the frame indicated in B. VIP immunoreactivity is found in nerve cell bodies in the ventral SCN (red) with nerve fiber projections in–and outside the SCN, many reaching the dorsal part of the SCN where the VPAC2 receptors (green) are present (D). In 3D reconstruction (Z-stack of 90 images) as indicated with frame in D of the ventral SCN, close appositions most likely representing synaptic appositions between VIP immunoreactive nerve fibers and VPAC2 receptor are indicated (blue in E). Arrows in E and F indicate VIP cells also expressing the VPAC2 receptor. PAC1 and VPAC2 receptor are expressed in separate neurons of the mouse SCN (G-L). The VPAC2 receptor (green) is widely distributed in the SCN with slightly higher occurence in the shell region (G). The PAC1 receptor (red) is also expressed in the SCN with a slightly higher occurrence in the retino-recipient part (H). In I both receptors are stained on the same section. High magnification and 3D analysis on a Z-stack consisting of 37sections in the mid-ventral SCN (marked with * in I) are shown in J-L. In K-L examples of a SCN neuron expressing the PAC1 receptor is indicated by a, a neuron expressing both the PAC1 and the VPAC2 receptor is indicated by b, and one neuron expressing the VPAC2 receptor is indicated with c. 3v; third ventricle, oc; optic chiasm. Scale bars: A; 50 μm, B and E; 20 μm, D; 100 μm, C;7 μm, F; 5 μm, G-I; 50 μm, J-L; 5 μm.

**Fig 5:**
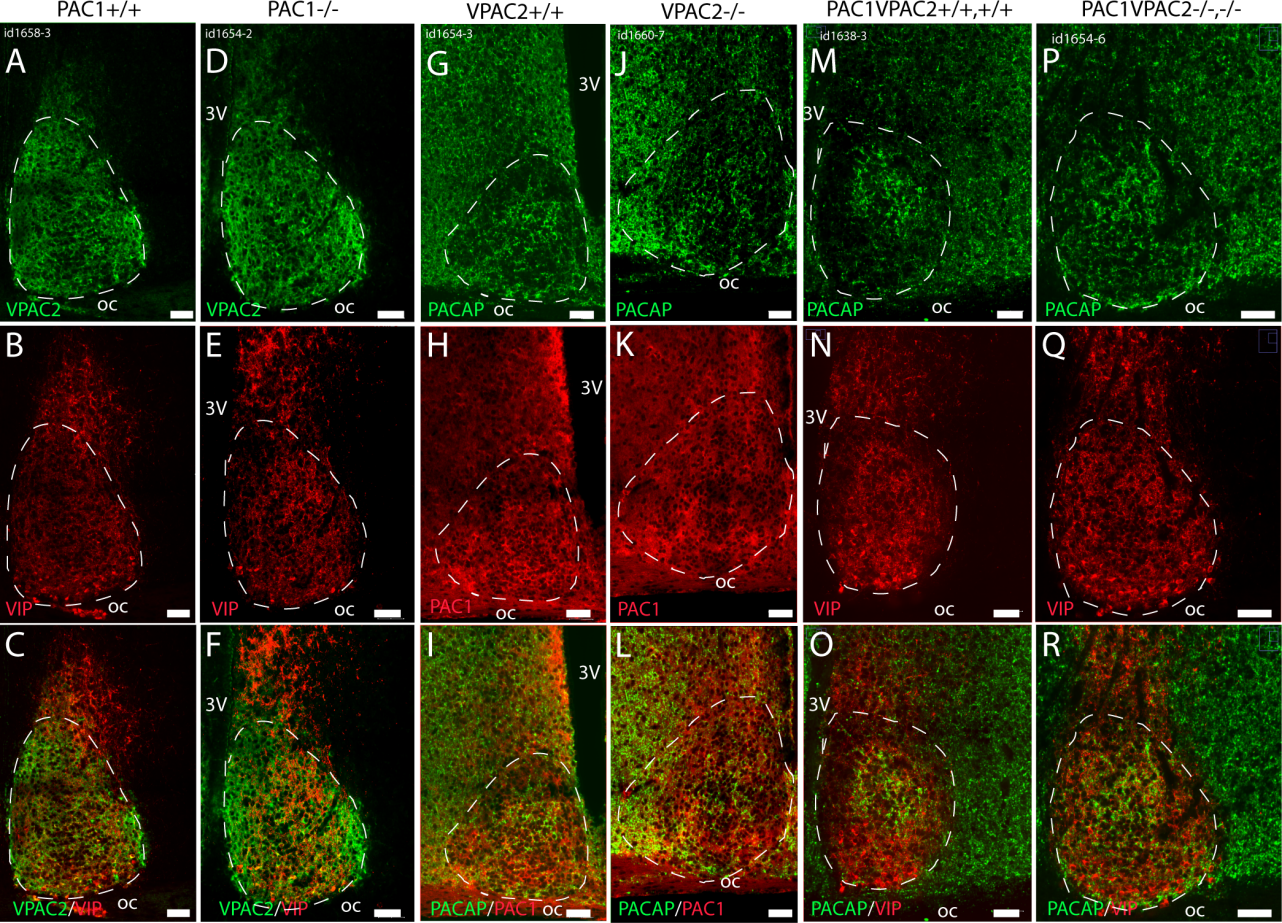
No compensatory changes are found in PAC1 –or VPAC2 receptor expression, or in the ligands (PACAP and VIP) in the SCN of mice lacking the reciprocal receptor when comparing wild type and PAC1 and VPAC2 knockout mice (A-L). PACAP and VIP expression (M-R) did not differ between wild type—and double mutant mice. Note PACAP immunoreactivity in many nerve fibers in the hypothalamus and distinct in the retino-recipient part of the mid SCN (M and P and O and R). 3v; third ventricle, oc; optic chiasm. Scale bars: A-R; 50 μm.

By immunohistochemistry PACAP immunoreactive nerve fibers were found in the SCN corresponding to the input from the RHT (Fig 4A and Fig 5G, 5J, 5M and 5P). Intense PACAP immunoreactivity was also found throughout the hypothalamus (Fig 5G, 5J, 5M and 5P). PAC1 immunoreactivity was found in neurons of the SCN and in close apposition to PACAP containing nerve fiber terminals with a slightly higher expression in the retino-recipient part of the SCN (Fig 4A–4C and Fig 5I and 5L). From stacks of images using high magnification, 3D-reconstuction and deconvolution, close appositions most likely representing synaptic appositions were found between PACAP-immunoreactive (ir) nerve fibers and PAC1-ir located in the membrane of SCN neurons (Fig 4B–4C).

### Localization of VIP and VPAC2 receptor in the mouse SCN

Immunohistochemical staining demonstrated VIP containing neurons of the ventral SCN and in a dense network of nerve fibers projecting within the SCN and out of the nucleus (Fig 4D–4F and Fig 5B, 5E, 5N and 5Q). Within the SCN, VIP-ir nerve fibers were found in both the ventral retinorecipient part (core) and in the dorsal part (shell) (Fig 4D–4F and Fig 5B, 5E, 5N and 5Q). Intense VPAC2 immunoreactivity was found in the SCN with the highest expression in the shell region of the SCN (Fig 4D, Fig 5G and 5J). VIP containing nerve fibers were in all parts of the SCN found in close apposition to VPAC2 receptors located in the neuronal membrane of cell soma or proximal dendrites (Fig 4E–4F). From stacks of images using high magnification, 3D-reconstuction and deconvolution, close appositions most likely representing synaptic appositions were found between of VIP containing nerve fibers and the VPAC2 receptors located in the membrane of SCN neurons (Fig 4F). In the ventral SCN few VIP immunoreactive cells co-expressed VPAC2 immunoreactivity (Fig 4E and 4F).

### PAC1 and VPAC2 receptors are co-expressed on few SCN neurons

Double immunostaining of PAC1 and VPAC2 showed that (Fig 4G–4L) the PAC1 receptor was primarily expressed in cells corresponding to the retinal inputs (core) (Fig 4H) and VPAC2 receptor had the highest expression in neurons of the shell area (Fig 4G). Occurrence of both receptors on the same cells was sparse but a few examples in the mid/central SCN could be demonstrated in 3D reconstructed images (Fig 4L).

### No compensatory changes in the expression of peptide and/or receptor in PAC1-, VPAC2- and PAC1/VPAC2 deficient mice

We used immunohistochemical staining of PACAP, PAC1 receptor, VIP and VPAC2 receptor on sections through the SCN from wild type and single–and double mutant mice to evaluate whether the gene deficient mice showed sign of compensatory changes in expression. In neither PAC1 receptor–nor VPAC2 receptor deficient mice no visible changes in ligand (Fig 5B, 5E, 5G and 5J) or receptor expression (Fig 5A, 5D, 5H and 5K) were observed in the SCN. Also, no changes in expression of PACAP or VIP were found in double receptor deficient mice (Fig 5M–5R).

## Discussion

Light has great impact on physiology and behavior and is the most important “zeitgeber” used by the circadian system to stay entrained by the solar cycle [12]. Light also affects behavior directly, a process known as masking [25]. Masking can be considered as another control mechanism, which in addition to circadian control, shape behavior and physiology to the rhythmic changed on our planet. In nocturnal animals, light induces negative masking which during daytime may protect the animal against activity behavior exposing it for predators. PACAP is together with glutamate implicated in both light entrainment and in negative masking as neurotransmitter in the RHT [13, 16, 26]. Mice lacking the VPAC2 receptor lack circadian control but display rhythmic locomotor activity and stable core body temperature in a normal photoperiod depending on signals from the RHT. By investigating the PAC1-, the VPAC2- and the double mutant mice during different light regimes we provide evidence that despite structural similarities between PACAP and VIP as well as the PAC1 and the VPAC2 receptor, no common–or compensatory role seems to exist between the two peptide systems regarding physiology or gene expression. At the physiological–as well as at the behavioral level, the significant phenotype found in VPAC2 mutant mice is transferred to double mutant mice, which emphasizes the important and specific role of VPAC2 receptor signaling. Both genotypes become arrhythmic when placed in constant darkness and both genotypes display an almost inverted TP profile being more like a diurnal animal. VPAC2 receptor single mutant mice have been investigated in several laboratories. Overall, a similar circadian phenotype as in the present study has been reported, although some VPAC2 knock out mice are able to keep circadian rhythmicity even after several cycles in constant darkness [4, 5, 10, 27]. In a previous study on VPAC2 deficient mice we only occasionally found occurrence of circadian rhythmicity [5]. These studies on VPAC2 deficient mice support that VIP/VPAC2 signaling plays an essential role in the control of circadian activity in the SCN, promoting rhythmicity and synchronizing pacemaker neurons [28]. In both VIP and VPAC2 receptor deficient mice, RW activity is synchronized to the night, when animals are kept in FPP at high light intensity. This “masking” of a disrupted clock function in VPAC2 deficient mice is fully transferred to the double mutant mice. However, the present study shows that during a full photoperiod at low light intensity VPAC2- and double mutant mice display a not previously described phenotype in which RW activity onset is stably advanced by 6 h compared to wild type mice. This observation supports that RW activity in these animals is regulated by masking and that the light intensity plays a role. This may also explain a behavior observed in previous studies in which VPAC2KO mice kept in RW performed an unexplained “phase jump” when released into constant darkness [4, 5, 10, 27].

When comparing light regulated behavior in VPAC2- and double mutant mice a specific role of PAC1 receptor signaling seems to be disclosed. Masking behavior during low light intensity is mediated by PAC1 receptor signaling [16] and while VPAC2KO mice display two activity periods following the morning and the evening light pulse, double mutant mice display only one activity period following the morning light pulse. The “loss” of the evening activity period indicates a specific role of PAC1 receptor signaling during this period of the LD cycle. Comparing the RW activity in VPAC2- and double mutant mice during SPP, a one h light pulse has great impact on activity, which is significantly increased after lights off. While the SPP regime entrains intact animals to a full photoperiod, VIP deficient mice demonstrate two activity bouts following SPP [8]. In comparison, VPAC2 deficient mice display a classical negative masking response after light exposure corresponding to both “morning” and “evening” stimulation [25], whereas double mutant mice lack the “evening” response. Interestingly, “evening/early night” masking response was significantly compromised in PAC1 deficient mice [16]. VPAC2 knockout mice have been shown to display an altered gating to photic inputs [27] while constant light seems to stabilize clock cell synchronization in these mice [29]. This raises the possibility that PACAP and VIP signaling together are involved in the day/night setting. Such notion seems to be supported by observation of TP in the three genotypes. While the PAC1 deficient mice display TP cycle as wild type mice, single VPAC2 –and double mutant mice display an “diurnal-like” or inverted TP rhythm compared to PAC1 deficient–and wild type mice. The inverted phase of TP is observed in FPP as well as in SPP. However, TP phase seems to be less influenced by the light intensity, which could indicate that several mechanisms are involved in TP regulation. This may also account for the regulation of metabolism in mice lacking the VPAC2 receptor. Both metabolism and feeding behavior were reported to be phase advanced as TP in VPAC2 and VIP deficient mice and it was concluded that “light does not overtly mask these behaviors” [10]. The present observations suggest that VPAC2 receptor signaling plays a role not only for intracellular synchronization of SCN neurons to keep the same phase, but also plays a role via light signaling and the PAC1 receptor in shaping diurnality versus nocturnality.

By immunohistochemistry using newly raised antibodies against the PAC1 and the VPAC2 receptors, we addressed several issues related to the physiological observations of two peptide systems in the SCN. We first characterized the specificity of the two antibodies by 1) testing the occurrence on cell lines expressing the respective receptors as well as on control cell lines by immunohistochemistry and Western blotting. Both receptors were found mainly in the cell membranes and the extracted protein had size corresponding to the predicted proteins. 2) No immunostaining was found in neither of the respective knockout mice confirming that the staining represents the localization the PAC1 and the VPAC2 receptor, respectively.

The distribution of the PAC1 receptor protein is in full agreement with the localization of PAC1 mRNA distribution in the mouse brain [19] (see also Allen’s Brain Atlas: http://mouse.brain-map.org/gene/show/11304). As mentioned there is evidence that PACAP and the PAC1 receptor play a role in light entrainment of the SCN rhythm (reviewed in [17], see also [16, 26, 30–32]. Although previous studies have demonstrated the expression of the PAC1 receptor mRNA in the mouse SCN, localization of PAC1 receptor protein is demonstrated for the first time in the present study. According to the role of PACAP as neurotransmitter in the RHT, PACAP-ir fibers were found corresponding to the retinal innervation of the mouse SCN and the PAC1 receptor occurred in neurons in the SCN with the highest expression corresponding to the retinal terminal field. Furthermore, 3D analysis followed by co-localization provided evidence that the close apposition between the PACAP-ir nerve fibers and PAC1 receptor-ir represents synaptic innervation.

VPAC2 immunoreactivity in the SCN has been studied in mouse using commercial antisera [7]. As in the present study, VPAC2 immunoreactivity was found mainly in the cell membrane of neurons located in the shell region of the SCN and co-expressed in VIPergic neurons of the ventral SCN suggesting autoregulatory input from VIP containing cells in this part of the SCN. We found, however, that the majority of VPAC2 receptor expressing cells was located in the dorso-medial SCN. In this part of the SCN, the most dense intra-SCN innervation of VIPergic nerve fibers was observed and detailed 3D analysis followed by co-localization provided evidence that the close apposition between the VIPergic nerve fibers and VPAC2 receptor immunoreactivity represent synaptic innervation of the VPAC2 expressing neurons. Neurons in this part of the SCN, many of which express AVP, also harbors the clock genes Per1 and Per2 which display rhythmic expression, and our anatomical observations support the physiological role of VIP-VPAC2 receptor signaling in the SCN synchronizing the individual clock neurons in the SCN to a coherent rhythm [28].

Semi quantitative evaluations of the level of receptor and PACAP and VIP in the respective genotypes were performed by immunohistochemistry. This gave information of distinct expression and localization, which could not be obtained by other more quantitative analyses such as western blotting or other i.e. radioimmunoassays. Using IHC, we found no major changes in immunostaining of neither receptor protein nor ligands in the SCN of the different genotypes. These findings are in accordance with our physiological observations where we also found no signs of compensatory changes in physiology and behavior.

## Conclusion

Our data demonstrate that in mammals although being closely related at both ligand and receptor structure/sequence, PACAP/PAC1 receptor signaling is independent of VIP/VPAC2 receptor signaling and verse versa. Lack of either of the receptors causes no compensatory changes at neither the physiological nor anatomical level. By comparing circadian behavior and physiology of single and double mutant mice during different light conditions of FPP and SPP at high and low light intensity evidence was provided that PACAP/PAC1 signaling is important for light regulated behavior, VIP/VPAC2signaling for stable clock function and both signaling pathways may play a role in shaping diurnality versus nocturnality.

## Supporting information

S1 Fig Characterization of antibodies against the PACAP specific PAC1 receptor (PAC1) and the VIP receptor2 (VPAC2). (TIF)

S1 File Supporting information. (DOCX)
