# Supplementary figures and images for: PAC1- and VPAC2 receptors in light regulated behavior and physiology: Studies in single and double mutant mice
Source: PLoS One. 2017 Nov 20;12(11):e0188166. doi: 10.1371/journal.pone.0188166 (PMC5695784; doi:10.1371/journal.pone.0188166)

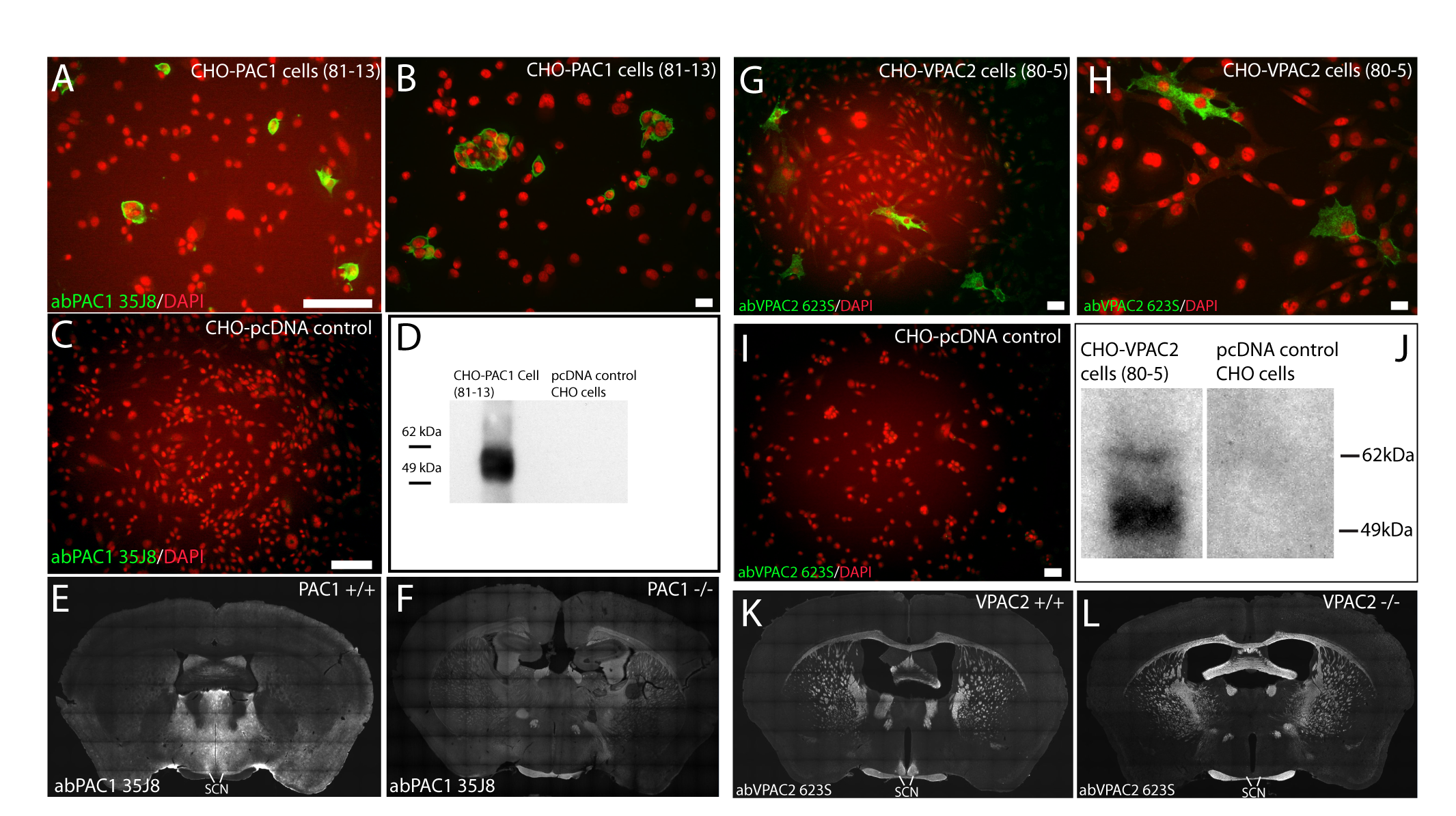

Supplement: S1 Fig — (TIF) [file pone.0188166.s001.tif]
