# Supplementary material for: PAC1- and VPAC2 receptors in light regulated behavior and physiology: Studies in single and double mutant mice
Source: PLoS One. 2017 Nov 20;12(11):e0188166. doi: 10.1371/journal.pone.0188166 (PMC5695784; doi:10.1371/journal.pone.0188166)
Supplement: S1 File — (DOCX) [file pone.0188166.s002.docx]

**Supporting information**

**PAC1 antiserum**

Immunostaining of the CHO-PAC1 expressing with antibody Ab35J8 showed cells with intense PAC1 immunoreactivity in the cell membrane while no immunostaining was obtain in the control cell line (S1 FigA-C). The anti-PAC1 antibody detected a band of approximately 62 kDa in PAC1 receptor expressing CHO cells (81-13) (S1l. Fig D) corresponding to the size of mouse PAC1 protein. Immunostaining of the mouse forebrain for PAC1 receptor reveal intense immunostaining in the membrane of neurons of the hypothalamus including the SCN (S1Fig E, Fig 4). Immunoreaction was absent in brain sections from PAC1 deficient mice (S1 Fig F).

**VPAC2 antiserum**

Immunostaining of the VPAC2 expressing CHO-cells with antibody Ab623S showed cells with strong immunoreactivity in the cell membrane while no immunostaining was obtain in the control cell line (S1 FigG, H, I). The VPAC2 antibody ab623S detected a band of approximately 55 kDa in CHO cells stable transfected with a VPAC2 receptor expressing plasmid (Clone 80-5) (S1 Fig J) corresponding to the size of the mouse VPAC2 protein. No immunoreactivity was found in the parental negative control CHO cells (S1Fig I-J). Immunostaining of the mouse forebrain for VPAC2 receptors showed strong immunostaining associated with the cell membrane of soma and dendrites in the SCN (S1 Fig K, Fig4). No signal was found in brain sections from VPAC2 deficient mice (S1FigL).

**S1 Fig**

Characterization of antibodies against the PACAP specific PAC1 receptor (PAC1) (A-F) and the VIP receptor2 (VPAC2) (G-L). PAC1 protein visualized by immunohistochemical staining (green) in CHO cells expressing the PAC1 receptor (A-B). No staining was found in control CHO cells (C). The PAC1 protein had the expected size close to 62 kDa when extracted from the overexpressing cell clone on Western blot (D). Immunostaining on mouse coronal sections at the level of the SCN demonstrated PAC1-IR in many hypothalamic areas including the SCN (E). No staining was found in mice lacking the PAC1 receptor (F). VPAC2 protein was visualized by immunohistochemical staining (green) in CHO cells expressing the VPAC2 receptor (G-H), while no staining was found in control CHO cells (I). The VPAC2 protein has the expected size close to 55 kDa when extracted from the overexpressing cell clone on Western blot (J). Immunostaining on mouse coronal sections at the level of the SCN demonstrated intense labeling of VPAC2-IR in the SCN (K). No staining was found in mice lacking the VPAC2 receptor (L). Scale bars: A; 100 µm , B; 25 µm , C; 100 µm ; G; 50 µm, H; 25 µm, I; 50 µm.
